# A method for improving the performance of gradient systems for diffusion-weighted MRI

**DOI:** 10.1002/mrm.21379

**Published:** 2007-10

**Authors:** Zoltan Nagy, Nikolaus Weiskopf, Daniel C Alexander, Ralf Deichmann

**Affiliations:** 1 Wellcome Trust Centre for Neuroimaging, Institute of Neurology, University College LondonLondon, UK; 2 Centre for Medical Image Computing, Department of Computer Science, University College LondonLondon, UK; 3 University Hospital, Brain Imaging CentreSchleusenweg 2-16, Frankfurt 1 Main, Germany

**Keywords:** diffusion tensor imaging, apparent diffusion constant, magnetic field gradient, fibre tracking, anisotropy

## Abstract

The MR signal is sensitive to diffusion. This effect can be increased by the use of large, balanced bipolar gradients. The gradient systems of MR scanners are calibrated at installation and during regular servicing visits. Because the measured apparent diffusion constant (ADC) depends on the square of the amplitude of the diffusion sensitizing gradients, errors in the gradient calibration are exaggerated. If the error is varying among the different gradient axes, it will affect the estimated degree of anisotropy. To assess the gradient calibration accuracy in a whole-body MRI scanner, ADC values were calculated for a uniform water phantom along each gradient direction while monitoring the temperature. Knowledge of the temperature allows the expected diffusion constant of water to be calculated independent of the MRI measurement. It was found that the gradient axes (±x, ±y, ±z) were calibrated differently, resulting in offset ADC values. A method is presented to rescale the amplitude of each of the six principal gradient axes within the MR pulse sequence. The scaling factor is the square root of the ratio of the expected and observed diffusion constants. In addition, fiber tracking results in the human brain were noticeably affected by improving the gradient system calibration. Magn Reson Med 58:763–768, 2007. © 2007 Wiley-Liss, Inc.

The effect of diffusion on the NMR signal was first described in 1954 by Carr and Purcell (1). The method has received much attention since the incorporation of diffusion-encoding gradients into MRI sequences (2). Diffusion encoding is achieved by the application of magnetic field gradients (3, 4) and is particularly sensitive to gradient calibration errors. The gradients are usually calibrated by the vendors at installation and as part of the regular servicing. For example, a water phantom of a given diameter or one with fiducials at a known distance apart can be used for this calibration (5, 6). However, this accuracy is inherently limited by the spatial resolution of images acquired during the calibration. As the diffusion-encoding gradient amplitude is raised to the second power in the apparent diffusion constant (ADC) calculations, the percentage of error at the calibration stage is exaggerated further (7, 8). An additional problem may arise from the fact that the diffusion encoding gradients usually have higher amplitudes than the imaging gradients.

The diffusion coefficient is a well-defined physical quantity that is measurable by independent methods (i.e., not based on MRI). The so-called apparent diffusion constant (ADC) (8), which is obtained when MRI is used to measure the intrinsic diffusion coefficient, is expected to be an accurate and precise estimate. Most studies that intend to exploit ADC or anisotropy information obtained from diffusion-weighted images would either have to assume or ensure that the estimated ADC is accurate and the measured isotropy/anisotropy is unbiased by system performance (7).

Although the dependence of diffusion measurements on gradient performance has been discussed recently (9), previous work addressing gradient imperfections focused on correcting spatial nonlinearities instead of precise calibration of the gradient amplitudes (5, 6, 10, 11). Furthermore, these studies presented postprocessing corrections of the acquired images, which were based on the estimated distortion fields.

The present work outlines a procedure, which involves standard diffusion-weighted imaging on a water phantom, to determine whether the gradient amplitudes are miscalibrated. Should such a miscalibration exist, the resulting diffusion encoding *b*-value will be affected because it depends directly on the gradient amplitude (7, 8). To correct for this, a method is presented that rescales the gradient amplitudes. The proposed test of the calibration is simple to perform and the correction method is easy to implement at the pulse programming stage. Furthermore, this process is not limited by the spatial resolution of the images used for the calibration.

To demonstrate the impact of exact gradient calibrations on in vivo diffusion tensor imaging (DTI) studies, a healthy adult subject was scanned. Probabilistic index of connectivity (PICo) (12, 13) mapping was performed on all datasets and the gradient correction was found to have a noticeable effect.

## METHODS

### Theory

In a uniform water phantom one would expect an isotropic diffusion profile regardless of the method employed to measure this profile. However, in MRI, if the gradients are not properly calibrated and/or the controllers for setting up the positive or negative gradients do not perform identically, the resulting diffusion-weighting *b*-values will be biased. In turn the measured diffusion constant will be incorrect in the respective directions. This may even impart anisotropy erroneously when different gradient directions for encoding diffusion have different errors.

In the following, it is assumed that images are collected with two different *b*-values. Those with the higher *b*-value serve to encode the effects of diffusion while the others are used as reference.

The following variables will be used:

D^t^ true value of the ADC
D^P^ ADC as calculated from the experiments with erroneous *b*-value (b^P^ below)

For the measurements with high *b*-value:

b^R^ requested *b*-value
b^P^ *b*-value actually played out on the scanner
S^P^ diffusion-weighted signal due to the erroneous *b*-value b^P^

For the measurements with low *b*-value (reference images):

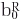
requested *b*-value for the reference image
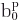
*b*-value actually played out for the reference image
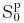
diffusion-weighted signal in the reference image due to the erroneous *b*-value 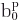

If the gradients are not properly calibrated, the unknown *b*-values b^P^ and 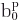 are played out. However, when the diffusion constant is calculated, the requested *b*-values b^R^ and 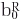 are still assumed, resulting in an incorrect value for the diffusion constant D^P^:

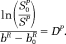
[1]
If the erroneous gradient performance was somehow identified and the incorrect *b*-values (b^P^ and 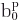) were hence known, the correct value of the diffusion constant could still be obtained according to:

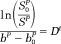
[2]
Note that the numerators on the left hand side of Eq. [1] and [2] are the same, leading to:

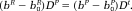
[3]
In both the Stejskal and Tanner (3) and the double refocused diffusion scheme (4) *b ∝ G*^2^, where G is the diffusion gradient strength. We correct for the miscalibrated gradients by a linear scaling factor so that:

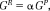
[4]
where G^R^ is the requested gradient strength and G^P^ is the gradient strength actually played out on the scanner. Furthermore, assuming that the miscalibrated gradient strength is the only cause for the discrepancy between b^R^ and b^P^ and between 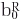 and 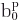, Eq. [4] implies that b^R^ = α^2^b^P^ and that 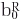 = α^2^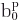. Substitution into Eq. [3] gives:

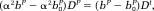
[5]
leading to:

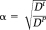
[6]
If D^t^ is obtained from an independent measurement (14), the factor α can be calculated for the individual gradient directions. For the subsequent experiments, these α values can be used to rescale the gradients and acquire data that will contain diffusion encoding of the desired *b*-values.

### Data Collection

All experiments were performed on a 1.5T whole-body scanner (Magnetom Sonata; Siemens Medical, Erlangen, Germany) operated with a body-transmit and a head-receive coil. All the experiments had the following common settings: TE = 90 ms, isotropic resolution = 2.3 mm, slices = 60, matrix size = 96× 96, field of view = 220 mm, twice refocused diffusion encoding according to Reese et al (4).

#### Phantom Experiments

Two different experiments were carried out using a spherical water phantom. Experiment 1 involved a diffusion-encoding sequence that consisted of 68 images, each with a unique diffusion direction. The *b*-value was 100 s/mm^2^ for the first seven images. These were used as reference in the calculations of the ADC. The *b*-value was 1000 s/mm^2^ for the other 61 images. The latter 61 directions were uniformly distributed on the surface of a hemisphere using the electrostatic minimization procedure (15). In experiment 2 the first seven images were identical to those in experiment 1 but were followed by 10 repetitions of each of the six principal gradient directions: (0, 0, ±1), (±1, 0, 0), and (0, ±1, 0). In both of the above scenarios the first seven images were averaged and used as the reference in the estimation of the ADCs along each of the 61 or 60 directions, respectively.

In each phantom experiment a 10 × 10 voxel region of interest was taken from the center of the middle slice of the volume (i.e., at isocenter). The ADC values reported in the Results section are the mean of the ADC values in these 100 voxels.

During these measurements a separate 750-ml water bottle was positioned close to the water phantom. The temperature of the water was measured in this container before and after each acquisition. The mean of the temperature before and after experiment 2 was used to calculate the true diffusion constant (D^t^) according to Mills (14). The thermometer was manufactured by Kane-May (model number KM330) with an accuracy of ±0.2% + 1°C. From the ADC values measured with MRI and the true diffusion constant (D^t^) the correction value (α) was determined for each of the six principle gradient directions (as given above) and subsequently used to modify the amplitude of the gradients within the pulse sequence program. Both experiments were repeated with this optimized sequence.

#### In Vivo Experiments

A healthy adult subject was also scanned to investigate the effect of the proposed gradient correction in vivo. Written informed consent was obtained prior to the experiment according to the guidelines of the local ethics committee. In this case the acquisition was pulse-triggered and three slices were acquired per heart beat.

To be able to compare the effects of interscan variability with that of the gradient correction, four sets of data were acquired. The first two datasets were acquired without any gradient modification. For the other two sets the gradients were rescaled using the α values obtained from the phantom experiment. In each case the scheme of experiment 1 was followed, acquiring seven images with the lower *b*-value followed by the 61 unique directions with the higher *b*-value. Postprocessing of the in vivo images was performed by one of the authors who was blinded to whether a given image set was collected with or without the corrected gradients. After realignment of the images in each set using the method of Bai et al. (16), which is based on Andersson and Skare (17), all 68 images were used to estimate the diffusion tensor by least squares fitting (18) to the log measurements. Fiber tracking was performed on the four sets of diffusion tensor images using Camino (13). This software is an open source toolkit for processing diffusion MRI data (http://www.cs.ucl.ac.uk/research/medic/camino). In addition, fractional anisotropy (FA) maps (19) were calculated.

A total of seven nonoverlapping seed regions were drawn in the splenium of the corpus callosum with sizes varying between 25 and 35 voxels. From each seed region PICo maps were generated (12) using default settings in the Camino software and a single diffusion tensor model for every voxel. The procedure ran 5000 streamlines from each of the seven seed regions in all the four datasets. In order to compare all the PICo maps the difference:

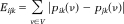
[7]
between all the possible pairs was computed, where *p*_*ik*_ and *p*_*jk*_ are the PICo maps from data sets *i* and *j* for seed region *k* and the sum is over every voxel *v* in the image *I*.

The above experiment was repeated in five other white matter regions with slight differences. Only five nonoverlapping seed regions were drawn in the genu of the corpus callosum. Only six nonoverlapping seed regions were drawn bilaterally in the cingulum and the corticospinal tracts. The size of seed regions varied between 10 and 19 voxels in the cingulum and between 10 and 25 voxels in the corticospinal tracts.

## RESULTS

### Phantom Experiments

**Figure 1:**
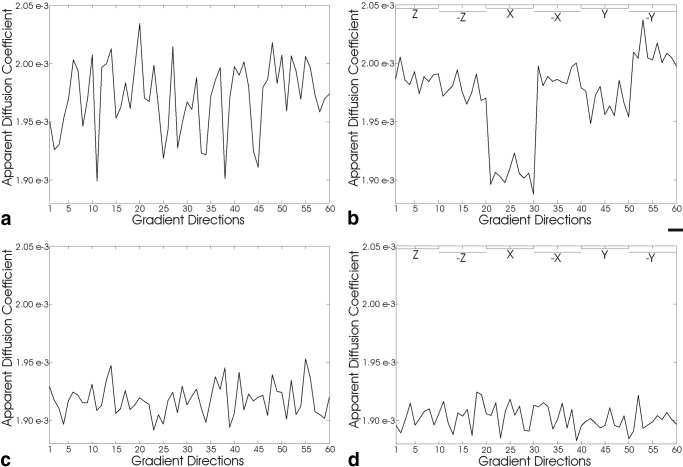
ADC values along different gradient directions. Two different experiments are described each performed without (top) and with (bottom) gradient corrections. The y-axis is scaled identically for all plots in units of mm^2^/s. In (**a**) and (**c**) the ADC values were calculated from seven images with b = 100 s/mm^2^ and 61 noncollinear directions distributed on the surface of a hemisphere with b = 1000 s/mm^2^ (for uniformity only 60 are shown). In (b) and (d) the ADC values were calculated from seven images with b = 100 s/mm^2^ and 10 images along both the positive and negative direction of each of the physical gradient axes with b = 1000 s/mm^2^ (see the top of each plot for indication of the gradient). The experiments were performed on a water phantom where isotropic diffusion is expected. **a**: Shows a high degree of variability in ADC values along the different directions. The variance is much higher than would be expected from the SNR of the images. **b**: Demonstrates that the variability in (a) is due in a large part to a systematic difference of ADC values along the different gradient axes. **c** and **d:** The results of the same two experiments as in (a) and (b) respectively after the gradient amplitudes were rescaled based on the methods described in this work (using Eq. [6]).

For experiment 1, ADC values along 60 different directions are shown in Fig. 1a. Contrary to the expectation of spatially uniform ADC values, there is a high degree of variability in this measurement. The percent difference between the minimum and maximum ADC values from this measurement is 6.9%. In contrast the mean coefficient of variation of the 60 regions of interest was 4.3%, leading to a standard error of the mean of about 0.4%.

**Figure 2:**
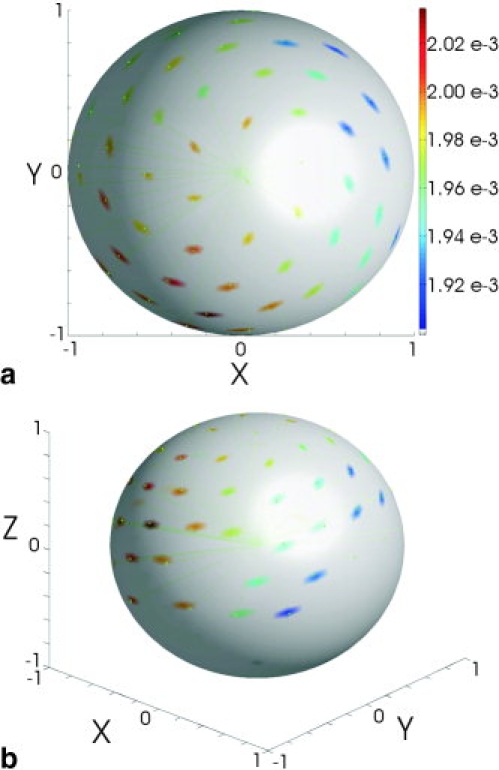
Three-dimensional display of ADC values in a common diffusion-weighted experiment. The data from Fig. 1a is plotted on the surface of a sphere. The tip of each of the gradient directions is color coded with the measured ADC value along that direction in units of mm^2^/s. **a:** Displays a view looking down the positive z axis. Note the large variability in ADC values at any given y coordinate when moving along x between ±1. In contrast there is much less variability along y at any given x coordinate. This observation is confirmed by the experiment, in which only ±x, ±y, and ±z gradients are used to encode diffusion (see Fig. 1b). **b:** Shows the same data as in (a) but from a side view.

For further analysis the ADC values were color-coded and plotted on the surface of the sphere along the respective directions of their acquisition (Fig. 2). This display demonstrates that the variability is spatially smooth and indicates discrepancies between positive and negative gradient directions along all gradient axes. The discrepancy is most severe for the x gradient axis.

The results of experiment 2 are plotted in Fig. 1b, which confirms the dependence of ADC values on the chosen gradient axis. The percent difference between the mean of the 10 positive and the mean of the 10 negative directions were 0.57% for the z-gradient, 4.3% for the x-gradient, and 2.2% for the y-gradient. The average temperatures were 18.2°C, 18.1°C, 18.25°C, and 18.25°C for the experiments in Fig. 1a–d, respectively. From experiment 2 in Fig. 1b, the expected value of the ADC was calculated to be 1.90 × 10^−3^ mm^2^/s (14). Using this value for D^t^ in Eq. [6] the gradients were individually rescaled (Table 1). More significant digits were kept than would be justified by the precision of the temperature measurement (see Discussion for reasons).

**Table 1:** Factor α for Each of the Six Principal Gradient Directions

Direction	α	% Change
+z	0.9776	2.2
−z	0.9804	1.2
+x	0.9990	0.0
−x	0.9776	2.2
+y	0.9831	1.2
−y	0.9726	2.7

Figure 1c and d shows the results for the corresponding experiments with the corrected gradient amplitudes. The variation in the ADC values along different gradient directions is noticeably reduced. Furthermore, measured ADC values are closer to the expected value.

### In Vivo Experiments

Table 2 shows the differences, calculated from Eq. [7], among all the possible pairs of PICo maps for the seed regions in the splenium of the corpus callosum (standard deviations [SDs] are given in parentheses). The difference between two PICo maps was always smaller if the PICo maps were generated from data sets that were either both acquired with the original gradients or both with the rescaled gradients. On the other hand these differences were all larger when one PICo map was generated from images without rescaling the gradients and the other PICo map was generated from images with rescaled gradients.

**Table 2:** Differences Between All the Possible Pairings of the Four PICo Maps Generated From Seed Regions in the Splenium of the Corpus Callosum*

	With rescaling #1	With rescaling #2	Without rescaling #1	Without rescaling #2
With rescaling #1	**0 (0)**	**191.2 (28.1)**	**272.4 (59.6)**	276.0 (54.1)
With rescaling #2		**0 (0)**	**260.3 (37.9)**	266.1 (38.2)
Without rescaling #1			**0 (0)**	**186.5 (48.0)**
Without rescaling #2				0 (0)

* For any comparison the mean and standard deviations (in parentheses) are given based on PICo maps that were generated using the seven nonoverlapping seed regions.

**Figure 3:**
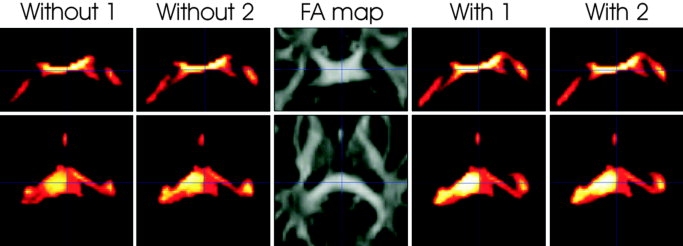
Comparison of PICo maps based on datasets with and without gradient rescaling. The same region is displayed in all five image sets. The top row is a coronal view and the bottom row is an axial view of the splenium of the corpus callosum. The middle plot displays the FA map. The two image sets to the left of the FA map are PICo maps based on image sets which were collected without correcting the gradients. To the right of the FA map are PICo maps based on image sets which were collected after the gradients were rescaled according to Eq. [6]. The value of each pixel is between zero and one and represents a probability of connectivity. The two PICo maps with the rescaled gradients show a higher degree of connectivity. [Color figure can be viewed in the online issue, which is available at www.interscience.wiley.com.]

Figure 3 shows all four PICo maps for one of the seven seed regions in the splenium of the corpus callosum. To help orientation the FA map of the corresponding region is also shown. Clearly the effect of the gradient corrections outweighs the effect of interscan variability. A *t*-test comparison was made between all repeats of the four pairs of PICo maps that were based on one image set with and one image set without rescaled gradients and all repeats of the other two pairs of PICo maps that were based on image sets that were both either with or without rescaled gradients. In other words the *t*-test was used to compare the 24 experiments (leading to the entries in normal font) with the 12 experiments (leading to the two entries in bold font). The results of this test show that repetition of scans causes less variability in PICo maps than does gradient correction (descriptive *P* < 10^−6^). There is therefore a significant effect of rescaling the gradients.

A similar experiment using seed regions within the genu of the corpus callosum and the right corticospinal tract provided similar results (descriptive *P* < 0.05). PICo maps from seed regions in the left corticospinal track did not show significant differences (*P* ≈ 0.1). Furthermore, none of the comparisons of PICo maps for the seed regions in the cingulum bundles showed significant differences. That is, in this region interscan variability was comparable in magnitude to the effect of rescaling the gradients.

## DISCUSSION

The present work shows that ADC measurements can be significantly biased by gradient miscalibration (in the order of a few percent). Furthermore, a method for correction is presented that is based on ADC measurements within a uniform water phantom and allows the amplitudes of the three gradient axes to be rescaled. This value is then compared to the expected diffusion constant at the given water temperature. The square root of the ratio between these two values provides the estimate of the gradient amplitude miscalibration. This factor can be used to rescale the gradients within the pulse sequence program. Although the inverse of this factor can also be useful as a postprocessing correction step for erroneous *b*-values, the focus in this work is on correcting the gradient amplitudes on the acquisition side. In that case the need for a correction in postprocessing software is eliminated.

Should this correction be required for a given scanner, it is likely that the rescaling factors will be unique to that machine. The actual values of these factors depend on a combination of hardware characteristics and the method and accuracy of the calibration used at installation or at the latest service by the manufacturer. The hardware characteristics are not likely to change significantly over time—unless of course the gradient coils are replaced, the amplifiers are changed or other major replacement takes place, which may affect gradient performance. After any such work the correction factor should be calculated with Eq. [6]. The recalibration may be necessary even after the regular servicing of the magnet depending on the reproducibility of the gradient calibration procedure.

Note that all the calculations presented assume that the amplitude of the gradient is solely responsible for the nonuniform ADC values along the different gradient directions. This assumption is supported by the successful correction of the systematic bias in the ADC values which exist without this rescaling (Figs. 1b and 2). However, it may be possible that deviations in the gradient ramp times are involved. Therefore further refinement may be possible.

Furthermore, it is possible that deviations of the gradient strength from the nominal value and DC offsets may contain nonlinear components. In addition, the *b*-value is affected by the interaction between the imaging- and diffusion-encoding gradients (20) since in principle all gradients contribute to the diffusion weighting. Therefore, calibrations as presented here should be performed for the *b*-value and imaging gradients that are going to be used in the actual experiment.

The calibration method presented here is based on diffusion measurements at the isocenter. Further deviations of the ADC value may be expected within off-center voxels if the gradients suffer from spatial nonlinearity. This effect, as well as a method of correction, will be thoroughly addressed in a future study.

The calibration procedure presented here helps to both remove erroneous anisotropy and to provide the expected diffusion constant. For the latter of these two the precision of the temperature measurement determines the precision of the calibration. In the Results section it was noted that in the recalibration of the gradients more significant digits were taken than permitted by the precision of the thermometer. The reason for this is that even if the true ADC value is missed, the erroneous anisotropy can be removed by scaling the gradients to the same value.

The method described here only corrects the diffusion-encoding gradients. The imaging gradient amplitudes of the sequence are unaffected. Neither will gradient amplitudes be affected in other sequences on the same scanner.

The human subject was scanned to investigate the effects of gradient rescaling in vivo and it was found that for most seed regions the changes in PICo maps due to gradient correction exceeded the normal interscan variability. However, when PICo maps were generated from seed regions in the cingulum, the gradient rescaling induced differences on the order of interscan variability. One explanation for this may be that this fiber tract runs predominantly in the anterior posterior direction while the biggest miscalibration of gradients was observed in the left-right direction. Another possible explanation could be reduced statistical power as the fiber tracks are inherently shorter than those in the other investigated regions, namely the corpus callosum and the corticospinal tract. It is notable that PICo maps that were based on data with corrected gradients showed a larger extent of connectivity. This suggests a better definition of fiber tracts (Fig. 3). However, fiber tracking based on diffusion imaging lacks a gold standard. Accordingly, we cannot claim that the rescaling improved the results.

Furthermore, neither is it claimed here that every investigation involving diffusion imaging will suffer from the miscalibrated gradient systems. It is, however, important to realize that the potential for erroneous results is always present if the gradient correction is neglected.

## CONCLUSION

Even if the conventional gradient calibration procedure is successfully carried out, the results of diffusion imaging experiments may still be significantly biased. We have developed and validated a correction method that improves the accuracy of ADC measurements with MRI by a more precise calibration of the gradient amplitudes. This calibration can improve the results of studies that rely on ADC measurements such as fiber tracking.
